# *Patagomaia* could be a gondwanatherian

**DOI:** 10.1038/s41598-024-78400-8

**Published:** 2024-11-19

**Authors:** Hans P. Püschel, Agustín G. Martinelli, Sergio Soto-Acuña, Alexander O. Vargas

**Affiliations:** 1 Millenium Nucleus on Early Evolutionary Transitions of Mammals, Santiago, Chile; 2 Red Paleontológica U-Chile, Laboratorio de Ontogenia y Filogenia, Departamento de Biología, Facultad de Ciencias, Universidad de Chile, Santiago, Chile; 3 CONICET-Sección Paleontología de Vertebrados, Museo Argentino de Ciencias Naturales “Bernardino Rivadavia”, Av. Ángel Gallardo 470, C1405DJR Buenos Aires, Argentina

**Keywords:** Palaeontology, Phylogenetics

**arising from**: N. R. Chimento et al.; *Scientific Reports *10.1038/s41598-024-53156-3 (2024).

In a recent contribution, Chimento et al.^1^ described fragments of the pelvis and hindlimb of a Late Cretaceous mammal from the Campanian–Maastrichtian Chorrillo Formation (Southern Patagonia, Argentina). They named a new genus and species, *Patagomaia chainko*, and assigned it to Theria, representing, in their opinion, the definitive first record of this clade in the Late Cretaceous of South America (other alleged therians were discussed by Rougier et al.^2^ who considered their assignment ambiguous). Here, we argue that an important alternative was not properly assessed: *Patagomaia* may be a gondwanatherian, a clade whose presence in South America is well-documented. Chimento et al.^1^ carried out a single phylogenetic analysis that included a gondwanatherian: *Adalatherium hui*^3^, from the Late Cretaceous of Madagascar, which is the only gondwanatherian for which associated, almost complete postcranial remains are known. Upon comparing *Patagomaia* and *Adalatherium*, we have found that all putatively therian characters of *Patagomaia* mentioned by Chimento et al.^1^ are either present in *Adalatherium*, or cannot be assessed in the latter. Further, several characters were incorrectly scored in the phylogenetic analyses of Chimento et al.^1^. When these scores are corrected, there is no longer support for therian affinities; instead, gondwanatherian affinities are found to be more plausible.

Among the alleged therian traits of *Patagomaia* mentioned by Chimento et al.^1^, an important character is the shape of the femoral head. Mammaliaforms may or may not have a femoral head with a neck: among those that do have a femoral neck, it can be short and incipient, or long with a well-offset femoral head as in Theria. *Adalatherium* shows a short and incipient femoral neck^6^. We do not find any significant differences in this character between *Patagomaia* and *Adalatherium* (Fig. 1). Nevertheless, Chimento et al.^1^ scored this character for *Patagomaia* as long with a well-offset femoral head (Character 64). In addition, a long and well-offset femoral head appears in other non-therian lineages, such as multituberculates^4,5^.

**Fig. 1:**
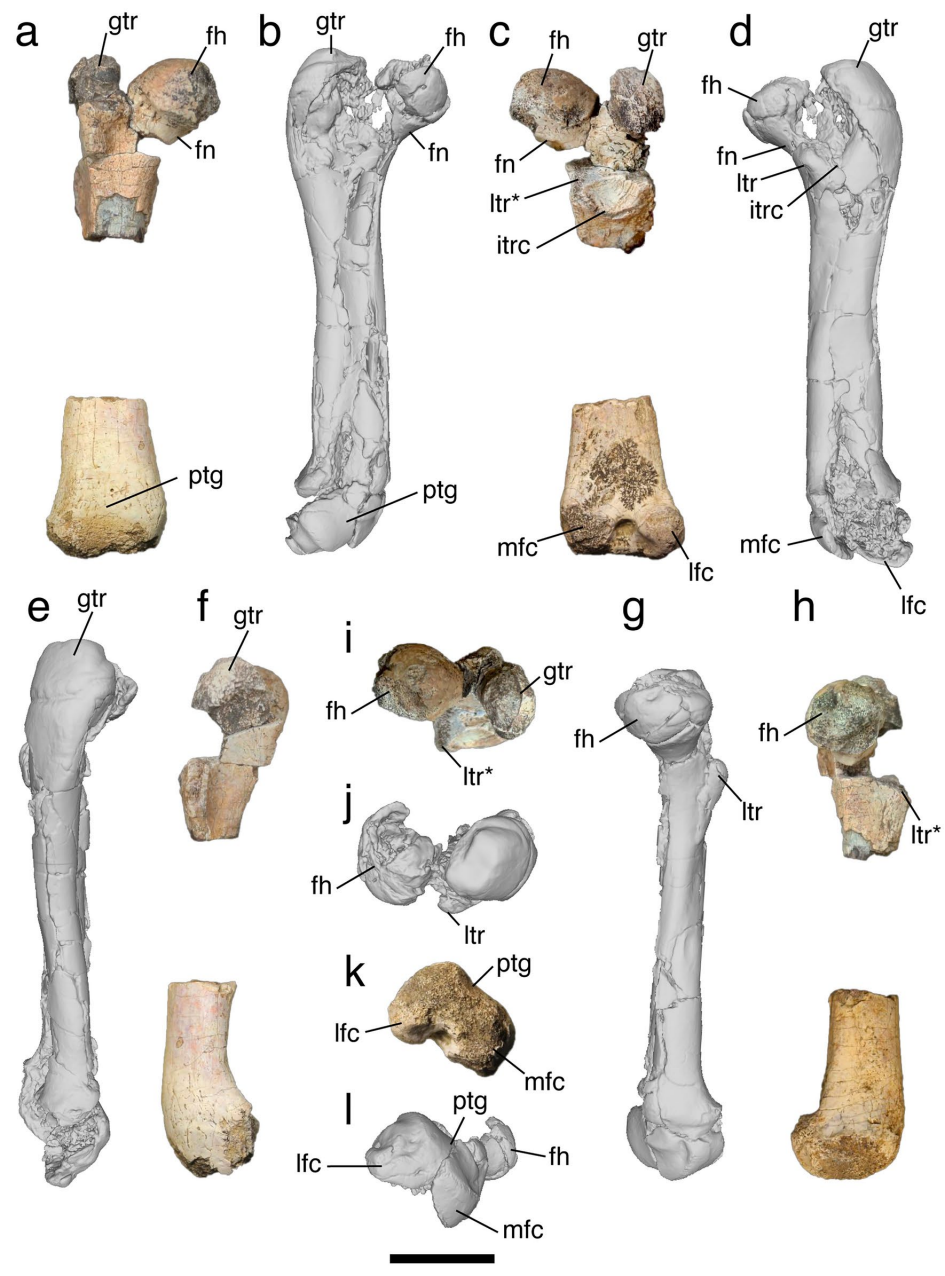
Right femora of *Patagomaia* (MPM-PV-23365) and *Adalatherium* (UA 9030; left femur mirrored). Femur of *Patagomaia* in anterior (**a**), posterior (**c**), lateral (**f**), medial (**h**), proximal (**i**), and distal (**k**) views. Femur of *Adalatherium* in anterior (**b**), posterior (**d**), lateral (**e**), medial (**g**), proximal (**j**), and distal (**l**) views. **fh**, femoral head; **fn**, femoral neck; **gtr**, greater trochanter; **lfc**, lateral femoral condyle; **ltr**, lesser trochanter; **mfc**, medial femoral condyle; **itrc**, intertrochanteric crest; **ptg**, patellar groove. Scale bar = 2 cm.

Other alleged therian-like characters of *Patagomaia* mentioned by Chimento et al.^1^ are the position of the lesser trochanter on the ventromedial or ventral side of the shaft (Character 68); an angle of about 55° formed between the femoral head and the main axis of femur (Fig. 1); a well-defined and wide trochanteric fossa, distally bounded by a crescent-shaped crest; a robust greater trochanter that is anteroposteriorly broad, and projected posteriorly reaching the same level as the femoral head; and a deeply excavated intercondylar fossa along the posterior surface of the shaft of the tibia. We found that all of these features are present in *Adalatherium.* The fact that so many similarities exist in pelvis and hindlimb structure between therians and gondwanatherians likely reflects locomotor-related homoplasies^6^. Other potentially therian-like characteristics of *Patagomaia* cannot be confidently assessed in *Adalatherium*, due to preservational issues of its only known specimen^6^. These characters include a pubis, ischium, and ilium strongly fused in the acetabular region; a therian-like acetabulum with a complete rim lacking a dorsal emargination (Character 57); a distal end of the femur that is transversely narrow, with proximo-distally low distal condyles nearly symmetrical in size and shape; a thick and prominent intercondylar ridge that is obliquely oriented and distally delimited by a pit-like concavity; and a tibia with symmetrical proximal facets for articulation with the femoral condyles. In addition, the bone histology features of *Patagomaia* cannot be compared to those of *Adalatherium* due to the lack of published, comparable information in the latter taxon.

We also found problems in the scoring of additional characters that may have affected their results (Table 1). Chimento et al.^1^ performed three phylogenetic analyses, using three different matrices^3,7,8^. From these matrices, we focus only on the matrix of Krause et al.^3^ as it is the only one of the three matrices that includes gondwanatherians in addition to therians. Therefore, this is the only matrix that allows to properly test the alternative hypothesis proposed here. Character 74 refers to the patellar groove of the femur, which was scored as absent in *Patagomaia* by Chimento et al.^1^. However, in distal view it is possible to observe that a shallow but distinct groove is present in *Patagomaia* (Fig. 1k), which is similar to the condition described for *Adalatherium*^6^ (Fig. 1l). In fact, Chimento et al.^1^ explicitly discuss the presence of this patellar groove in *Patagomaia*, and further highlighted it as “pg” in some of their figures (e.g., Fig. 1; the figures, unfortunately, lack a key for their abbreviations). We therefore corrected the score of the patellar groove as present. As a consequence, character 75 can now be scored, which refers to the mediolateral contour of the patellar groove (which is flat, as in *Adalatherium*; Fig. 1). Scored characters 59 and 79 refer to structures that are not preserved in the described specimens of *Patagomaia*, so we changed their scores to missing data (?).

**Table 1:** Differences in the character scoring of *Patagomaia* in Chimento et al.^1^ and this study.

Character	Chimento et al.^1^	This study
59: Pelvis, Ischiatic tuberosity, size	Small or absent (0)	?
64: Femur, Neck, degree of development	Distinct and long (1)	Incipient and short (0)
74: Femur, Patellar groove, presence	Absent (0)	Present (1)
75: Femur, Patellar groove, mediolateral contour	Non applicable (–)	Flat (0)
79: Fibula, Contact with femur, presence	Absent (1)	?

‘?’ represents missing data.

The features used in the generic diagnosis of *Patagomaia* should also be absent in *Adalatherium,* but this is not the case. The presence of a femur with a subspherical head showing a well-defined fovea capitis, a distal end of the femur with nearly symmetrical distal condyles and reduced epicondyles (Fig. 1), and an intercondylar ridge delimiting a deep fossa at the distal end of the femur, are all present in *Adalatherium*. The possibility that these characters are general traits of Gondwanatheria cannot be ruled out. Chimento et al.^1^ mentioned that the poorly preserved iliopubic eminence of *Patagomaia* (broken at its tip) “appears to have been robust” and thus different from *Adalatherium*. However, this is only a guess regarding the size of an unpreserved structure.

With these considerations in mind, we incorporated the new character scorings (Table 1) into the matrix of Krause et al.^3^ and ran parsimony (equal weights and implied weights) and Bayesian analyses (see Methods in Supplementary Information [SI]). The strict consensus tree of the equal weights parsimony analysis now recovers *Patagomaia* in a polytomy alongside gondwanatherians, as well as therians (Fig. 2a). Unlike the analysis in Chimento et al.^1^, we additionally used implied weights parsimony and Bayesian analyses, which are widely accepted approaches to further explore the tree space^9,10^. These did not recover any support for therian affinities, but instead, both approaches recovered *Patagomaia* as a gondwanatherian (Figs. 2b and S1 in SI). The inclusion of Bayesian analysis alongside parsimony is valuable and quickly becoming a new standard in phylogenetics^9^. Importantly, simulations have shown that in instances where a large amount of data is missing (as in the case of *Patagomaia*), Bayesian analysis produces less error in tree estimation when compared to parsimony^11^. To further test the phylogenetic affinities of *Patagomaia* we scored this taxon in another recently updated matrix^12^, which includes the gondwanatherians *Adalatherium* and *Vintana*, and 34 therians. Although this exact matrix was not available at the time of publication of the Chimento et al.^1^ study, a previous iteration of the matrix^13^ was available for them. As in the Krause et al.^3^ matrix, we recovered *Patagomaia* in a polytomy with many mammals in equal weights parsimony analysis (Fig. S2 in SI), whereas in the implied weights parsimony and the Bayesian analyses, *Patagomaia* was recovered as a gondwanatherian, in a polytomy alongside *Adalatherium* and *Vintana* (Figs. S3 and S4 in SI).

**Fig. 2:**
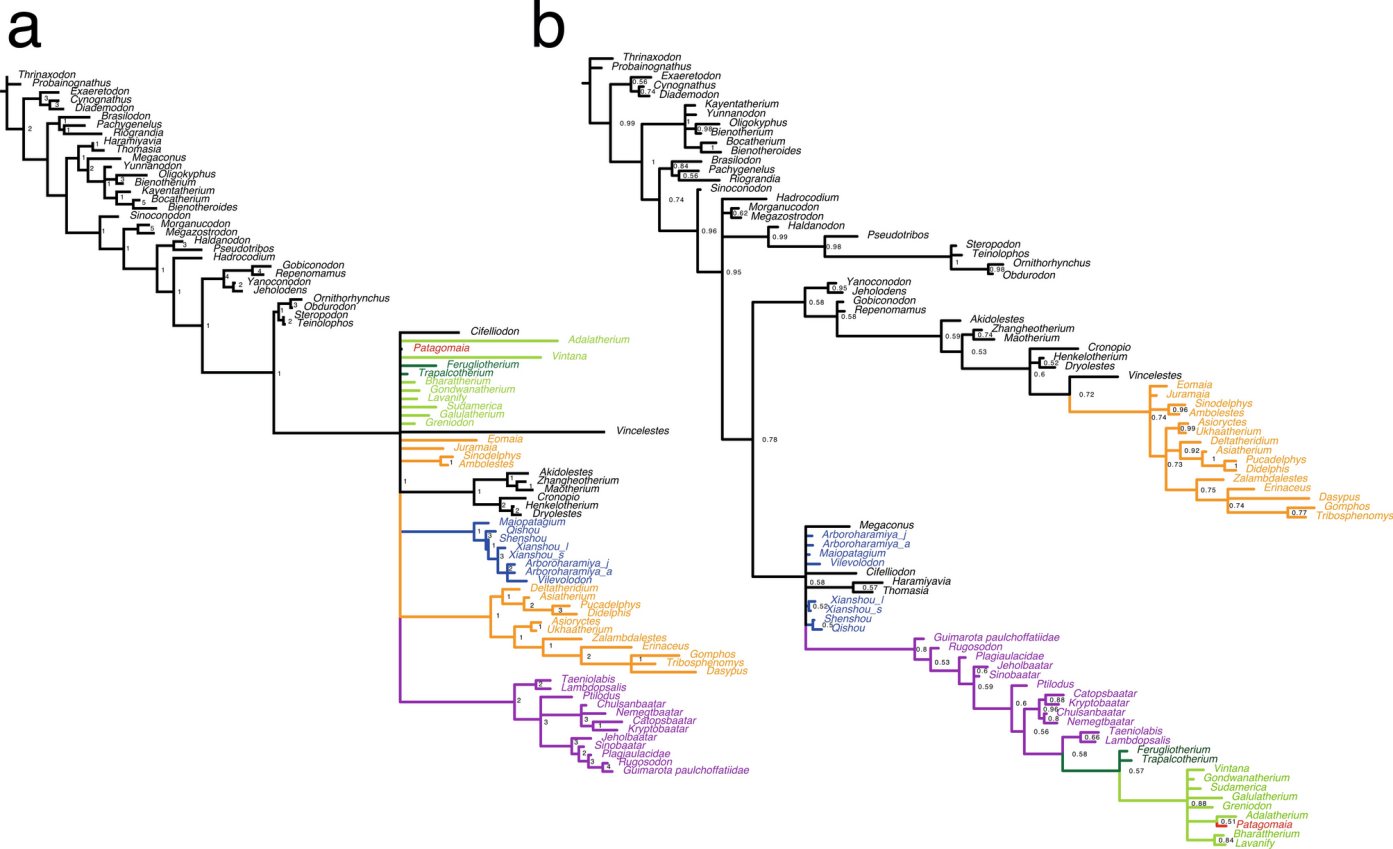
Parsimony and Bayesian phylogenies. (**a**) Strict consensus tree of the phylogenetic analyses including new scorings for *Patagomaia* (Table 1). We found 280 trees with 2305 steps. The consistency index was 0.303 and the retention index was 0.703. Node support is indicated with absolute Bremer support. (**b**) 50% majority rule tree of the Bayesian analysis. Node support is indicated with Bayesian posterior probabilities. None of these phylogenies suggests a particularly close affinity between *Patagomaia* and therians. In both trees, *Patagomaia* is highlighted in red, ferugliotheriids in dark green, gondwanatherians excluding ferugliotheriids (*Galulatherium*, *Adalatherium* and sudamericids) in light green, multituberculates in purple, euharamiyidans in dark blue, and therians in orange.

When working with very partial information, rigorous anatomical comparisons are the appropriate level of analysis to generate preliminary hypotheses on phylogenetic affinities. Phylogenetic analysis can be carried out, but information for only a few postcranial characters as in *Patagomaia* (representing about 2% of scorable characters in the matrix) implies the results are unstable and can be easily altered by a few changes. Although our results support gondwanatherian affinities for *Patagomaia* as the best hypothesis, its phylogenetic affinities can only be confidently tested with more informative remains. For now, the evidence for therian affinities is far from compelling. A great diversity of Cretaceous mammals existed in South America, but most species are known only from dental materials^2^. In these conditions, to avoid potential taxon redundancy^14^, we suggest greater caution when naming a new taxon solely based on postcranial fragments with no associated teeth. Unlike therians, the presence of definitive gondwanatherians in Patagonia is well-documented, and dental remains at a nearby coeval site (the Chilean side of Southern Patagonia) show how the gondwanatherian *Magallanodon* achieved large body sizes in the same size range as *Patagomaia*. *Patagomaia* could represent such a large gondwanatherian, if not *Magallanodon* itself.

## Supplementary Information

Supplementary Information.
