# Supplementary material for: Patagomaia could be a gondwanatherian
Source: Sci Rep. 2024 Nov 19;14:28632. doi: 10.1038/s41598-024-78400-8 (PMC11576905; doi:10.1038/s41598-024-78400-8)
Supplement: Supplementary file 1 — Supplementary Information. [file 41598_2024_78400_MOESM1_ESM.docx]

**Supplementary Information for:**

***Patagomaia* could be a gondwanatherian**

Hans P. Püschel^1,2*^, Agustín G. Martinelli^1,3^, Sergio Soto-Acuña^1,2^, Alexander O. Vargas^1,2^

^1^ Millennium Nucleus Early Evolutionary Transitions of Mammals

^2^Red Paleontológica U-Chile, Laboratorio de Ontogenia y Filogenia, Departamento de Biología, Facultad de Ciencias, Universidad de Chile, Santiago, Chile

^3^CONICET-Sección Paleontología de Vertebrados, Museo Argentino de Ciencias Naturales “Bernardino Rivadavia”, Av. Ángel Gallardo 470, C1405DJR CABA, Argentina

*Corresponding author: hpuschelr@gmail.com

**List of content:**

1. **Institutional abbreviations**
2. **Methods**
3. **Matrices employed in the phylogenetic analysis and character scorings**
4. **Maximum parsimony analyses**
5. **Bayesian analyses**
6. **Additional results from the maximum parsimony and Bayesian analyses**

**1)** **Institutional abbreviations**

**MPM-PV**, Museo Padre Molina, Río Gallegos, Santa Cruz, Argentina; **UA**, Université d’Antananarivo, Antananarivo, Madagascar.

**2) Methods**

**a) Matrices employed in the phylogenetic analysis and character scorings**

From the three matrices, employed by Chimento et al.^1^, we only used the matrix of Krause et al.^2^ as this matrix contains a good representation of gondwanatherians and therians, being the only one that allowed us to test the hypothesis that *Patagomaia* might be a gondwanatherian instead of a therian. The other two matrices^3,4^ do not include any member of Gondwanatheria and therefore are inadequate for testing this hypothesis. We modified some of the scorings of Chimento et al.^1^ that we considered to be incorrect (see main text and Table 1). The character scores that we used in the analysis for *Patagomaia* in the Krause et al.^2^ matrix are the following:

*Patagomaia* ????????????????????????????????????????????????????????11?1??1011111?000101??????????????????????????????????????????????????????????????????????????????????????????????????????????????????????????????????????????????????????????????????????????????????????????????????????????????????????????????????????????????????????????????????????????????????????????????????????????????????????????????????????????????????????????????????????????????????????????????????????????????????????????????????????????????????????????????????????

In addition, to further test the phylogenetic affinities of *Patagomaia* we employed the matrix of Mao et al.^5^, which contains a sample of 128 mammaliamorphs including the gondwanatherians *Adalatherium* and *Vintana*, and 34 therians. As this matrix contains both gondwanatherians and therians, it is adequate for testing our hypothesis concerning the potential gondwanatherian affinities of *Patagomaia*. It is important to mention that Mao et al.^5^ matrix has a different taxon sampling and character list from the Krause et al.^2^ matrix, allowing the independent testing of alternative phylogenetic hypotheses. We scored a total of 13 characters in the Mao et al.^5^ matrix, which were all the characters that the remains of *Patagomaia* allowed us to score. The characters and character scores of *Patagomaia* are the following:

344. Acetabular dorsal emargination: (0) Open (emarginated); (1) Closed (with a complete rim). ***Patagomaia*: 1.**

345. Sutures of the ilium, ischium, and pubis within the acetabulum: (0) Present; (1) Fused. ***Patagomaia*: 1.**

355. Inflected head of the femur set off from the shaft by a neck: (0) Neck absent and head

oriented dorsally; (1) Neck present, head spherical and inflected medially. ***Patagomaia*: 1.**

356. Femur neck degree of development: (0) Incipient and short; (1) Distinct and long. ***Patagomaia*: 0.**

357. Fovea for the acetabular ligament on the femoral head: (0) Absent; (1) Present. ***Patagomaia*: 1.**

358. Orientation of the greater trochanter: (0) Directed dorsolaterally; (1) directed dorsally.

***Patagomaia*: 1.**

359. Greater trochanter of femur: (0) Continuous with femoral head; (1) Separated from femoral head by distinct notch. ***Patagomaia*: 1.**

360. Level of greater trochanter relative to femoral head: (0) mid-level of femoral head; (1) top level of femoral head. ***Patagomaia*: 1.**

361. Position of the lesser trochanter: (0) On medial side of the shaft; (1) On the ventromedial or ventral side of the shaft. ***Patagomaia*: 1.**

362. Size of the lesser trochanter: (0) Large; (1) Small to absent. ***Patagomaia*: 1.**

364. Femur patellar groove presence: (0) Absent (0); (1) Present. ***Patagomaia*: 1.**

365. Femur patellar groove mediolateral contour: (0) Flat; (1) Concave. ***Patagomaia*: 0.**

366. Proximo-lateral tubercle or tuberosity of the tibia: (0) Large and hook-like; (1) Indistinct; (2) Fused to fibula. ***Patagomaia*: 1.**

All the character scores that we used in the analysis for *Patagomaia* in the Mao et al.^5^ matrix are the following:

*Patagomaia* ???????????????????????????????????????????????????????????????????????????????????????????????????????????????????????????????????????????????????????????????????????????????????????????????????????????????????????????????????????????????????????????????????????????????????????????????????????????????????????????????????????????????????????11?????????10111111?101?????????????????????????????????????????????????????????????????????????????????????????????????????????????????????????????????????????????????????????????????????????????????????????????????????????????????????????

**b) Maximum parsimony analyses**

We used TNT v.1.6^6^ for running the maximum parsimony analyses in both matrices^2,5^, setting the outgroup as *Thrinaxodon*. We conducted a "New Technology" driven search with sectorial search, ratchet, drift, and tree fusing, using default settings for each search algorithm, and finding the minimum length tree or best score 10 times. Once completed, we performed on the obtained trees an additional “traditional search” with the tree bisection and reconnection (TBR) branch-swapping algorithm. After completion, a strict consensus tree was computed from all the obtained most parsimonious trees (MPTs). In addition, considering that some authors (e.g.,^7,8^) advocate the use of implied weights instead of equal weights in matrices with an important degree of homoplasy, we ran the analyses again following almost the same procedure, but this time using implied weights with concavity (k) value of 12. This concavity value is relatively mild against homoplastic characters and has returned better results than stronger concavity values (e.g., k = 3) in simulated and empirical datasets^7^. Finally, the absolute Bremer supports (BS) for the nodes were calculated, and the consistency index (CI) and the retention index (RI) were estimated with the “STATS.RUN” script provided by TNT.

**c) Bayesian analyses**

The Bayesian analyses were performed in both matrices^2,5^ in MrBayes v3.2.7a^9^, setting *Thrinaxodon* as the outgroup. We defined one morphological partition with the Mkv model of morphological evolution^10^ using a lognormal distribution with eight rate categories to model rate heterogeneity across characters following Harrison and Larsson^11^. We ran the analyses using two independent runs of four chains for ten million Markov chain Monte Carlo (MCMC) generations, and then removed the first 25% of the samples as burn-in. After completion of the analyses, the deviation of the split frequencies was below 0.01 and the effective sample size of the parameters was >200. Additionally, both runs were visually inspected using Tracer v1.7.1^12^ to ensure that they reached convergence and stationarity. Finally, a 50% majority rule tree was computed and the posterior probabilities for the nodes (prob) were estimated.

**3) Additional results from the maximum parsimony and Bayesian analyses**


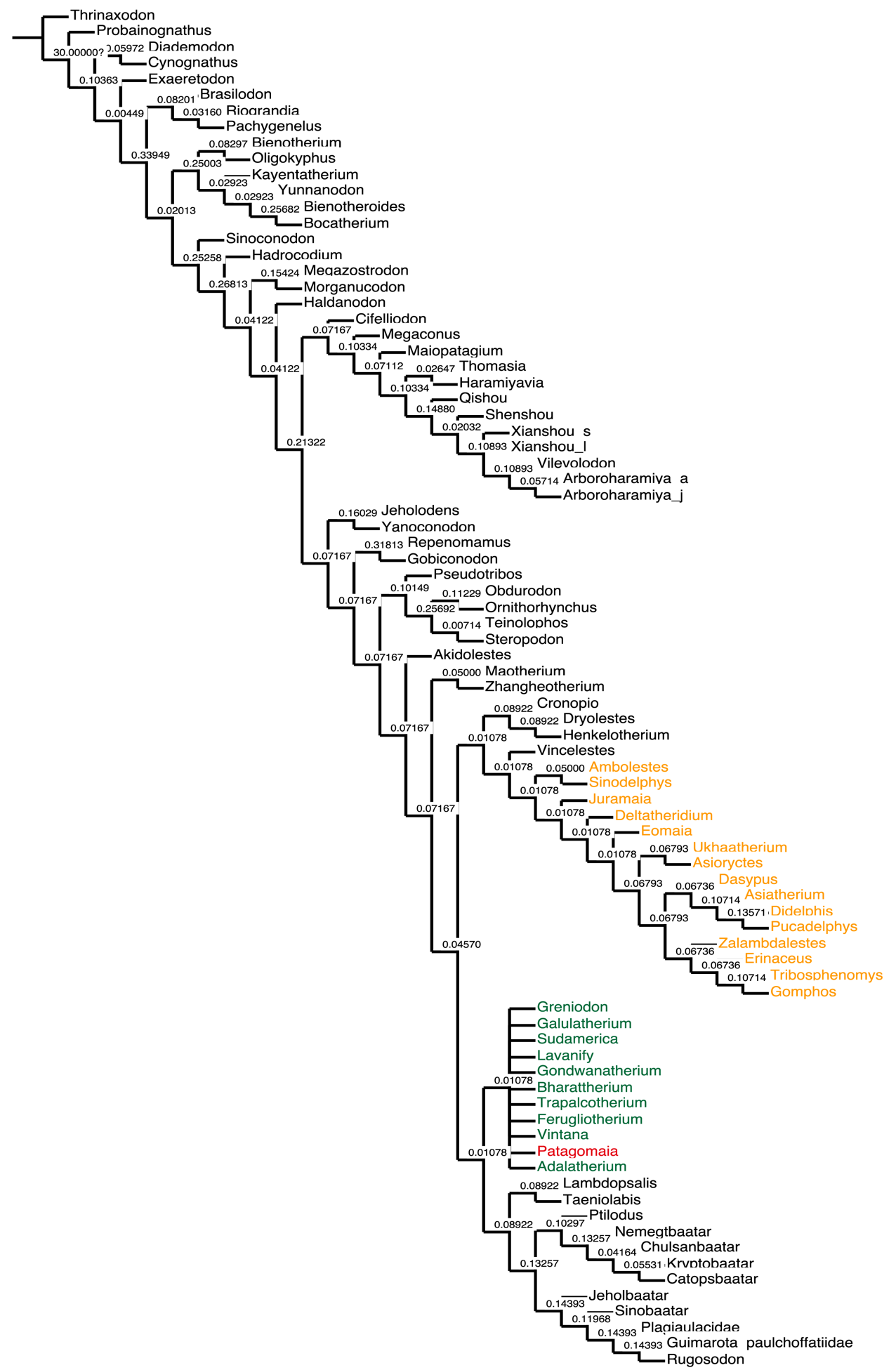


**Figure S1.** Strict consensus tree of the maximum parsimony analysis including new scorings for *Patagomaia* in the Krause et al.^2^ matrix (Table 1) and implied weights with a k = 12. We found 14 trees with 93.61217 steps. The consistency index was 0.3 and the retention index was 0.699. Node support is indicated with absolute Bremer support. *Patagomaia* is highlighted in red, gondwanatherians (including ferugliotheriids) in dark green, and therians in orange.


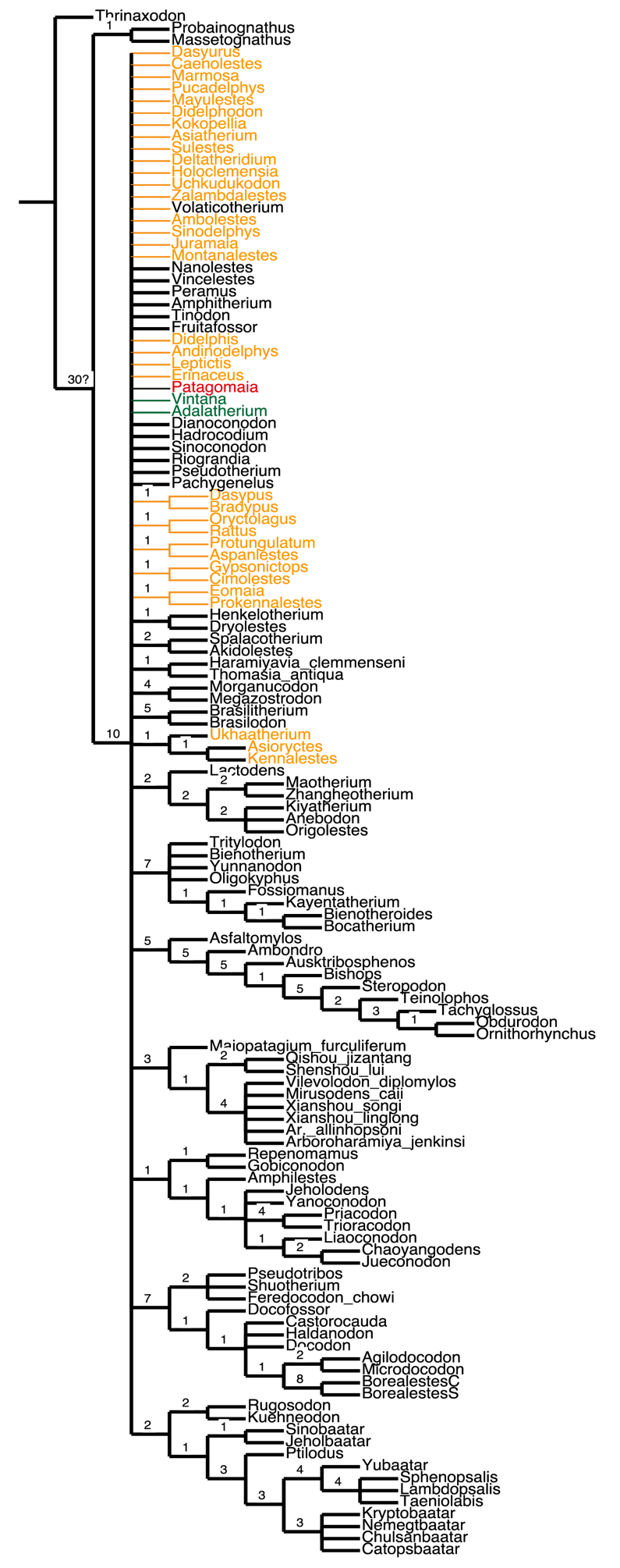


**Figure S2.** Strict consensus tree of the maximum parsimony analysis including scorings for *Patagomaia* in the Mao et al.^5^ matrix in an equal weights analysis. We found 100000 trees (memory overflown) with a best score of 2988. The consistency index and the retention index were not estimated as the memory was overflown with maximum parsimony trees. Node support is indicated with absolute Bremer support. *Patagomaia* is highlighted in red, gondwanatherians in dark green, and therians in orange.

**
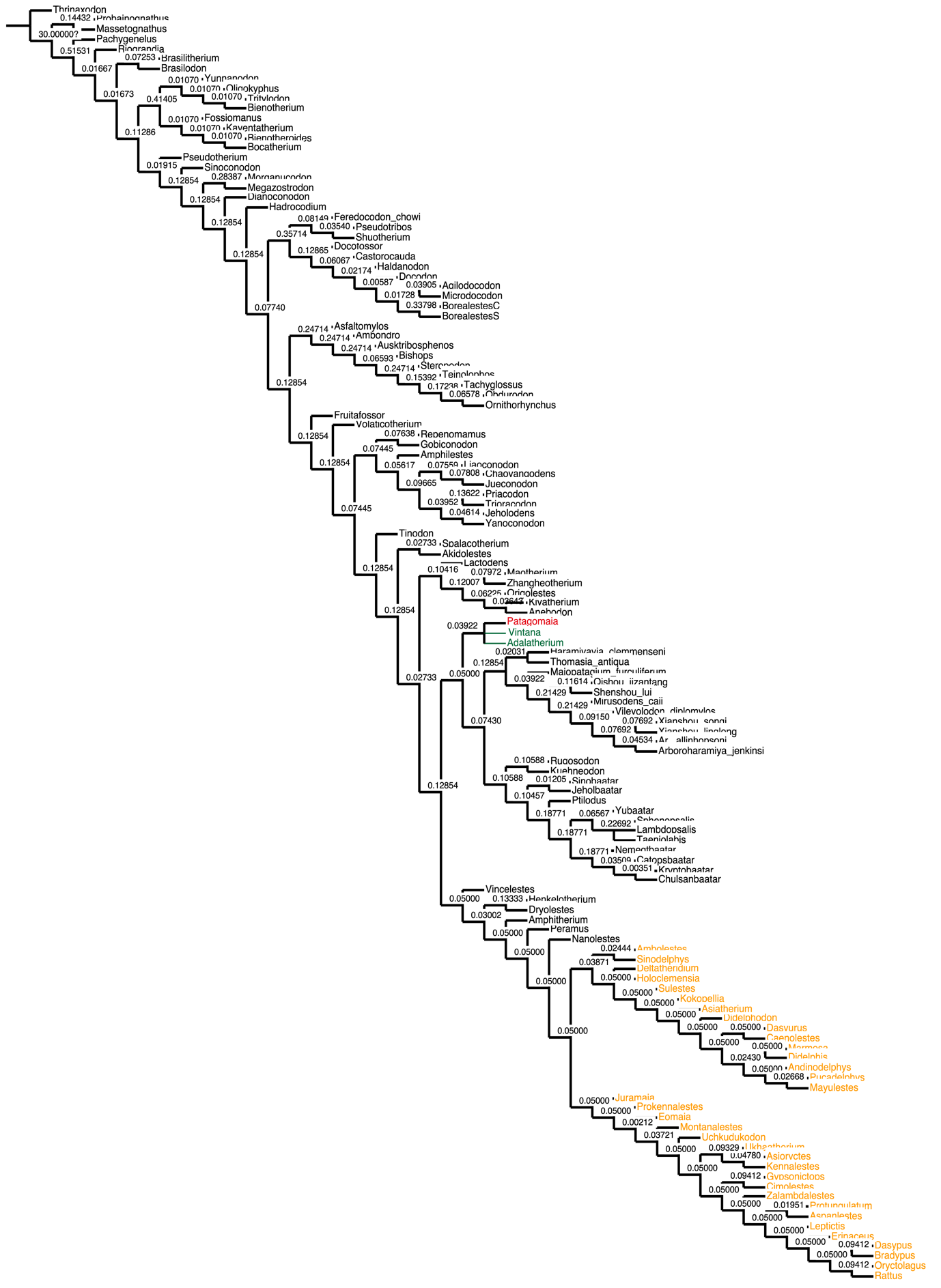
**

**Figure S3.** Strict consensus tree of the maximum parsimony analysis including new scorings for *Patagomaia* in the Mao et al.^5^ matrix and implied weights with a k = 12. We found 2 trees with 111.21658 steps. The consistency index was 0.324 and the retention index was 0.804. Node support is indicated with absolute Bremer support. *Patagomaia* is highlighted in red, and gondwanatherians in dark green.


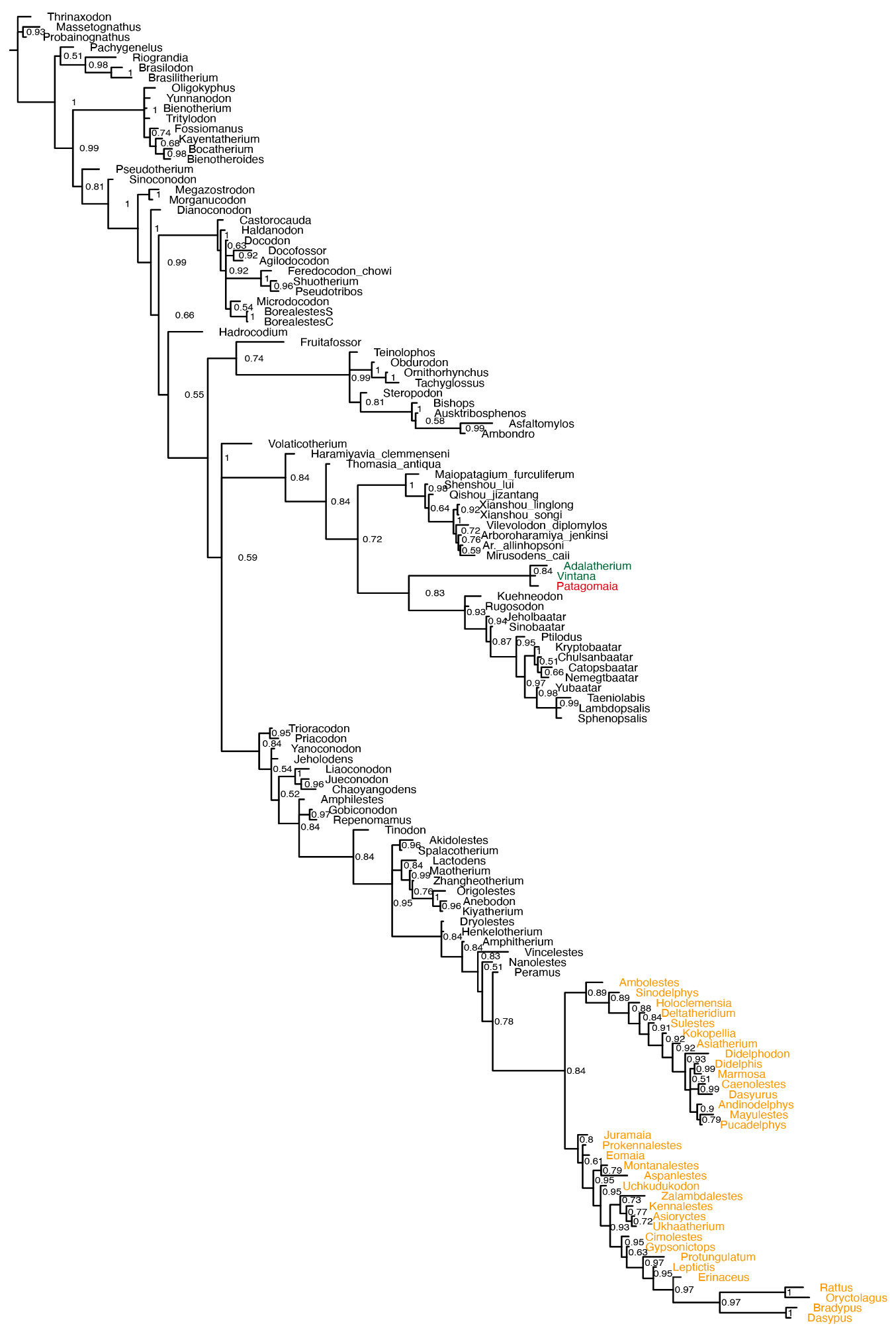


**Figure S4.** 50% majority rule tree of the Bayesian analysis including new scorings for *Patagomaia* in the Mao et al.^5^ matrix. Node support is indicated with Bayesian posterior probabilities. *Patagomaia* is highlighted in red, and gondwanatherians (including ferugliotheriids) in dark green.

**References**

1. Chimento, N. R. *et al.* A large therian mammal from the Late Cretaceous of South America. *Sci Rep* **14**, 2854 (2024).

2. Krause, D. W. *et al.* Skeleton of a Cretaceous mammal from Madagascar reflects long-term insularity. *Nature* **581**, 421–427 (2020).

3. Martinelli, A. G. *et al.* New cladotherian mammal from southern Chile and the evolution of mesungulatid meridiolestidans at the dusk of the Mesozoic era. *Sci Rep* **11**, 7594 (2021).

4. Zhou, C.-F., Bhullar, B.-A. S., Neander, A. I., Martin, T. & Luo, Z.-X. New Jurassic mammaliaform sheds light on early evolution of mammal-like hyoid bones. *Science* **365**, 276–279 (2019).

5. Mao, F. *et al.* Jurassic shuotheriids show earliest dental diversification of mammaliaforms. *Nature* **628**, 569–575 (2024).

6. Goloboff, P. A. & Morales, M. E. TNT version 1.6, with a graphical interface for MacOS and Linux, including new routines in parallel. **39**, 144–153 (2023).

7. Goloboff, P. A., Torres, A. & Arias, J. S. Weighted parsimony outperforms other methods of phylogenetic inference under models appropriate for morphology. *Cladistics* **34**, 407–437 (2018).

8. Goloboff, P. A., Carpenter, J. M., Arias, J. S. & Esquivel, D. R. M. Weighting against homoplasy improves phylogenetic analysis of morphological data sets. *Cladistics* **24**, 758–773 (2008).

9. Ronquist, F. *et al.* MrBayes 3.2: efficient Bayesian phylogenetic inference and model choice across a large model space. *Systematic Biology* **61**, 539–542 (2012).

10. Lewis, P. A likelihood approach to estimating phylogeny from discrete morphological character data. *Society of Systematic Biologists* **50**, 913–925 (2001).

11. Harrison, L. B. & Larsson, H. C. E. Among-character rate variation distributions in phylogenetic analysis of discrete morphological characters. *Systematic Biology* **64**, 307–324 (2015).

12. Rambaut, A., Drummond, A. J., Xie, D., Baele, G. & Suchard, M. A. Posterior summarization in Bayesian phylogenetics using Tracer 1.7. *Systematic Biology* **67**, 901–904 (2018).
